# Dr. Martin Barry on Fibre

**Published:** 1842-08-13

**Authors:** Martin Barry


*On Fibre.* By MARTIN BARRY, M. D.—Dr. Barry observes that in the mature blood-corpuscle there is often seen a flat filament already formed within it. In mammalia this filament is frequently annular. In birds, amphibia, and fishes, the filament is so long as to form a coil. This filament, he states, is formed of discs contained within the blood corpuscles. At the outer part of the coil, the filament, which is flat, often presents its edge, whilst in the centre there is generally found the unappropriated part of a nucleus. The nucleus, he says, in many cases resembles a ball of twine, being composed, at its outer part, of a coiled filament. In the invertebrata, examined by Dr. Barry, it is stated that the blood-corpuscle was likewise seen passing into a coil. This coil filament is described as being not only flat, but deeply grooved on both surfaces, and consequently thinner in the middle than at the edges, which were rounded, so that when seen edgewise the filament seemed to consist of segments.

It is mentioned that portions of the clot of blood consist of filaments having a structure identical with that found within the blood corpuscle. Dr. Barry states that he has seen the ring in the blood corpuscle of man, and the coil in that of birds and reptiles, unwind themselves into the straight and often parallel filaments of the clot. This appearance, he states, may be seen in blood placed under the microscope before its coagulation, and that filaments having the same structure may be met with in every tissue of the body.

He found the same filaments to exist in all vegetable structures, and he states that this, which he denominates a filament, is what has been usually described as a fibre. By a kind of apparent analogy, Dr. Barry endeavours to show that two spirals running in opposite directions, and interlacing at certain regular intervals, would produce an appearance which he could not distinguish from the simple filament, and he asserts that such an interlacement of two fibres would both produce a flat fibre, and also give it a grooved appearance. This, he asserts, is, in fact, the true structure of the fibre or *flat filament*, as he terms it. The edges of this filament present an appearance of segments ; but this he states is to be attributed to the consecutive curves of a spiral thread. The ultimate fibre or minutest filament, he states has this structure.

He hence asserts that the spiral arrangement is as general in the animal tissues as in vegetables. Nervous tissue, muscle, minute blood-vessels, and the crystalline lens are adduced in proof of his statements, and he even infers that spirals are as universal as the fibrous structure itself.

Dr. Barry states that the spiral form manifests itself very early, and refers to the corpuscles of blood in proof of this statement. Dr. Barry also states he observed, in the cartilages of the ear of a rabbit, the nucleus lying loose in its cell, resembling a ball of twine, being composed at its outer part of a coiled filament which it was giving off to weave the cell-wall.

Dr. Barry traces the formation of muscle out of cells, which, according to his observations, are derived from corpuscles of the blood. In this process he states he has observed the formation of a second order of tubes within the original tube; a peculiarly regular arrangement of discs within these second tubes; the formation, first of rings and then of spirals out of discs so arranged; the interlacing of the spiral; and the origin, in the space circumscribed by those, of spirals having a minuter size, which, in their turn surround others still more minute. The outer spirals are stated to enter into the formation of the investing membrane described by Schwann and Bowman. The inner spirals constitute what are usually termed fibrillæ, or which he terms a *flat filament*, and which, he asserts, is a compound structure. He asserts this fibril is not round and beaded, but flat and grooved, and, (if we understand the author aright) always "presents its edge to the observer," in voluntary muscles. In Dr. Barry's opinion, the dark longitudinal striæ are spaces, probably occupied by a lubricating fluid, between the edges of flat filaments, each filament being composed of two spiral threads, and the dark transverse striæ, rows of spaces between the curves of these spiral threads. He appears to attribute the contraction of a muscle to the contraction of the supposed spirals.

He states that the toothed fibre of the crystalline lens, described by Sir David Brewster, is formed of an enlarged filament, the projecting portions of the spiral thread becoming the teeth of that fibre.

The compound filaments are stated to be seen with peculiar distinctness in the blood-vessels of the arachnoid membrane. In connection with the spiral direction of the outer filaments in these vessels, Dr. Barry refers to the rouleaux in which the red blood discs are seen to arrange themselves under the microscope, as probably indicating a tendency to produce spiral fibres.—*Edinburgh Medical and Surgical Journal, July* 1, 1842.

